# An Examination of the Putative Role of Melatonin in Exosome Biogenesis

**DOI:** 10.3389/fcell.2021.686551

**Published:** 2021-06-08

**Authors:** Hassan Amini, Aysa Rezabakhsh, Morteza Heidarzadeh, Mehdi Hassanpour, Shahriar Hashemzadeh, Shahrouz Ghaderi, Emel Sokullu, Reza Rahbarghazi, Russel J. Reiter

**Affiliations:** ^1^Student Research Committee, Tabriz University of Medical Sciences, Tabriz, Iran; ^2^Cardiovascular Research Center, Tabriz University of Medical Sciences, Tabriz, Iran; ^3^Koç University Translational Medicine Research Center (KUTTAM), Istanbul, Turkey; ^4^Stem Cell Research Center, Tabriz University of Medical Sciences, Tabriz, Iran; ^5^Tuberculosis and Lung Disease Research Center, Tabriz University of Medical Sciences, Tabriz, Iran; ^6^Medical Faculty, Institute of Molecular Medicine III, Heinrich Heine University Düsseldorf, Düsseldorf, Germany; ^7^Department of Applied Cell Sciences, Faculty of Advanced Medical Sciences, Tabriz University of Medical Sciences, Tabriz, Iran; ^8^Department of Cell Systems and Anatomy, University of Texas Health Science Center at San Antonio, San Antonio, TX, United States

**Keywords:** melatonin, exosome biogenesis, interplay signaling pathways, cross talk, paracrine activity

## Abstract

During the last two decades, melatonin has been found to have pleiotropic effects via different mechanisms on its target cells. Data are abundant for some aspects of the signaling pathways within cells while other casual mechanisms have not been adequately addressed. From an evolutionary perspective, eukaryotic cells are equipped with a set of interrelated endomembrane systems consisting of intracellular organelles and secretory vesicles. Of these, exosomes are touted as cargo-laden secretory vesicles that originate from the endosomal multivesicular machinery which participate in a mutual cross-talk at different cellular interfaces. It has been documented that cells transfer various biomolecules and genetic elements through exosomes to sites remote from the original cell in a paracrine manner. Findings related to the molecular mechanisms between melatonin and exosomal biogenesis and cargo sorting are the subject of the current review. The clarification of the interplay between melatonin and exosome biogenesis and cargo sorting at the molecular level will help to define a cell’s secretion capacity. This review precisely addresses the role and potential significance of melatonin in determining the efflux capacity of cells via the exosomal pathway. Certain cells, for example, stem cells actively increase exosome efflux in response to melatonin treatment which accelerates tissue regeneration after transplantation into the injured sites.

## Introduction

**FIGURE 1:**
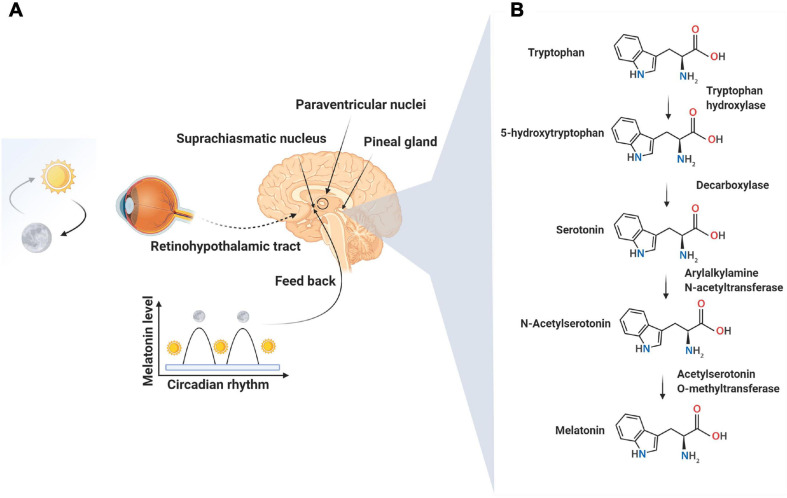
Neural control of melatonin synthesis in the pineal gland **(A)**. Melatonin, a well-known sleep-promoting molecule, is produced in the pineal gland as summarized in this figure. The secretion of melatonin is tightly controlled by suprachiasmatic and paraventricular nuclei in response to the light: dark cycle and the circadian clock mechanisms of the suprachiasmatic nuclei. The peak secretion of melatonin occurs at night time and reaches minimum levels at day. The synthesis of melatonin is a result of the enzymatic conversion of tryptophan to melatonin via the intermediate, molecule, serotonin **(B)**.

Numerous bioactivities and therapeutic effects of melatonin have been reported, especially within the last two decades. This hydrophobic molecule is produced by the pineal gland, specifically during the night, and in perhaps the mitochondria of all other cells (Tan et al., 2013; Reiter et al., 2020; Figure 1). Melatonin functions via well-described signaling pathways in host cells (Wei et al., 2020). When used by humans, melatonin is typically taken in the evening to improve sleep and, under some clinical conditions, also throughout the day (Castillo et al., 2020; Matar et al., 2021). By means of juxtacrine interactions, cells can transfer soluble biomolecules and factors to other cells at near and remote distances in a paracrine manner using nano-sized vesicles identified as exosomes (Whitham et al., 2018). Exosomes, which range from 40 to 200 μm, develop a suitable biological platform of biomolecule exchange and mutual crosstalk. Distinct intracellular mechanisms for sorting, trafficking, and abscission have been described, all of which are relevant to exosome biogenesis (Soekmadji et al., 2017). The presence of various genomic and proteomic factors inside exosomes makes them important agents that promote/inhibit certain signaling pathways once they reach their target cells (Minciacchi et al., 2015). Cells release the exosomes under physiological and pathological conditions in response to different factors. Whether the quantity and quality of exosomal cargo differ in different states is the subject of debate (Hooper et al., 2020). Research progress has led to a partial understanding of the intracellular mechanisms involved in exosome biogenesis. Several factors involving the regulation, synthesis, and abscission of exosomes require further studies. Various signaling cascades with multiple effectors are differentially regulated by endogenous or exogenous molecules which in turn influence exosome homeostasis (Bagheri et al., 2018). Within a cell, reciprocal crosstalk between the exosome-contained molecules and other signaling pathways regulate the paracrine capacity via the modulation of exosome biogenesis and abscission (Hassanpour et al., 2018a; Xu et al., 2018). Recent work has focused on the regulatory impact of melatonin on exosome biogenesis (Yoon et al., 2020). This review provides information related to the association of exosomes with melatonin signaling pathways. These pathways have been shown to determine and/or enhance the paracrine actions of melatonin.

## Exosome Biogenesis and Function

**FIGURE 2:**
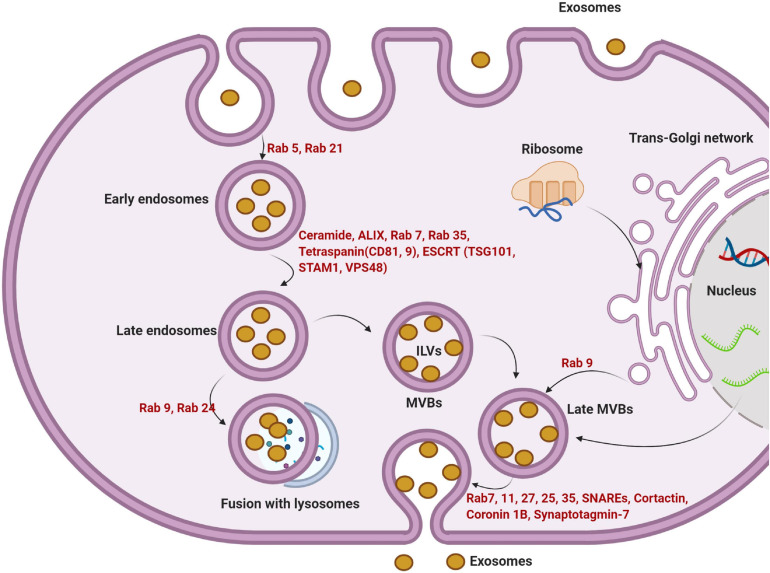
The multi-step intracellular maturation of exosomes is illustrated. For the initial internalization via endocytosis, GTPases including Rab5 and Rab21 play a significant role in the formation of early endosomes. The formation of late endosomes is done via engaging other important factors such as ceramide, ALIX, Rab7, 35, tetraspanins, and ESCRT complex. Due to the activity of other Rab types (Rab9 and 24), later endosomes are directed to lysosomes. Alternatively, late endosomes become MVBs. The invagination of the MVBs membrane forms numerous ILVs. Upon the release of ILVs to the ECM, they are thereafter referred to as exosomes. *Trans-*Golgi apparatus further supports an alternative way to form MVBs by the activity of certain GTPases such as Rab9. The fusion of later MVBs to the cell membrane is mediated by different effectors including Rab7, 11, 27, 25, 37, SNAREs, and other elements. ILVs, intraluminal vesicles; MVBs, multivesicular bodies; ESCRT, endosomal sorting complex required for transport; SNARE, soluble N-ethylmaleimide-sensitive factor attachment protein receptors.

Exosomes are small microvesicles of endosomal origin. Exosomes are functionally linked to multivesicular bodies (MVBs) and released after the fusion of MVBs with the cell membrane (Figure 2; Théry et al., 2009). Exosomes originate from terminal endosomes that are formed by the inward budding of the MVB membrane. In large MVBs, numerous intraluminal vesicles (ILVs) harboring exosomes are obvious (Minciacchi et al., 2015). Of note, ILVs are enriched in various cytosolic components such as soluble proteins, DNA, RNA, or microRNA. The receptor-mediated fusing of MVBs with the cell membrane releases the ILVs, now known as exosomes, into the extracellular matrix (ECM) (Valadi et al., 2007; Sahu et al., 2011). The complex protein machinery consists of four separate proteins including ESCRT-0, -I, -II, and -III and constitute the endosomal sorting complex required for transport (ESCRT) which accounts for MVB formation, vesicle budding, and cytosolic component cargo sorting (Figure 2; Record, 2014). The integrity of ESCRTs and complementary proteins is central to exosome biogenesis (Hurley, 2015). All the ESCRT subunits act via an ubiquitin-dependent pathway. The ubiquitin-binding subunits of ESCRT-0 sequester the ubiquitinated proteins at specialized domains of the endosomal membrane. Following the sequestration of target molecules, ESCRT-I and -II join the complex to develop a high-affinity recognition domain for the ubiquitinated proteins. The addition of the final subunit, ESCRT-III, causes the invagination of the lipid membrane and isolation of ILVs inside the MVB. Vps4 protein provides the energy required to separate buds from the MVB to form ILVs after the addition of ESCRT-III to the ESCRT-0, -I, and -II complexes (Figure 2; Ageta and Tsuchida, 2019). It is postulated that ESCRT machinery may influence exosomal quantity, size, and major cargo protein (Colombo et al., 2013).

The apparent specificity and function of exosomes have been determined. They are known to not only harbor exhausted biomolecules but also may contain specific therapeutic molecules with the ability to alter the function of target cells in relation to the immune response, antigen presentation, multiple signaling transduction actions in distant cells far from the original host cell (Huang-Doran et al., 2017; Hessvik and Llorente, 2018). The critical roles of exosomes have been implicated in multiple biological phenomena including important functions in tissue growth and development (Yáñez-Mó et al., 2015). However, direct evidence for the exact mechanism of action of exosomes on target cells is lacking. Using an N-ethyl maleimide-sensitive agent, an attachment protein receptor (SNARE) and membrane-bound Rab-GTPase, mainly Rab27, 11, and 35, were identified. Via these elements, MVBs are directed to the plasma membrane for the release of ILVs, exosomes, into the extracellular matrix (ECM) (Figure 2; Rezaie et al., 2018).

Evidence points to the paracrine and autocrine activity of released exosomes (Fitzner et al., 2011; Colombo et al., 2014). How released exosome contents act on specific distant cells in an autocrine manner rather than interacting with neighboring cells requires further investigation. The autocrine activity likely occurs in response to conditions when the host cells require stimulation or to recycle cargo protein (Xu et al., 2018). Numerous studies have explored the exosome cellular uptake and intracellular trafficking in acceptor cells. Several lines of evidence show that exosome influx is achieved through different pathways including direct fusion with the host cell membrane, receptor/ligand interaction, and internalization. Macropinocytosis, phagocytosis, and endocytosis are non-specific internalization mean for the transfer of exosomes into the cytosol (Mulcahy et al., 2014).

The rate of endocytosis increases when the generation of filopodia and lamellipodia is initiated. The distribution of cell membrane via cytoskeletal remodeling provides a platform to determine their uptake by different cells (Kamerkar et al., 2017). The existence of CD47 on the exosome surface mediates the “do not eat me” signal and helps them to evade internalization into the target cells (Kamerkar et al., 2017). CD47 also has an essential function to increase exosome transit time in the biofluids and blood (Zheng et al., 2020). Unlike the internalization pathway, the receptor/ligand interaction relies on the specific binding of ICAM-1 at the exosome surface with the LFA-1 receptor on the target cell plasma membrane (Segura et al., 2005). Data suggest the existence of specific molecules on the exosome’s surface that determine exosome internalization (Huang-Doran et al., 2017; Hessvik and Llorente, 2018). Whether receptor/ligand interaction, fusion, and the internalization pathway are dominant in exosome uptake under certain conditions remain undefined.

## Melatonin Biogenesis and Mechanism of Action

N-acetyl-5-methoxytryptamine, known as melatonin, is produced in the pineal gland and likely in the mitochondria of all other tissues; this ubiquitous molecule has highly pleiotropic effects (Claustrat and Leston, 2015). Pineal melatonin secretion into the blood and cerebrospinal fluid is linked to the light/dark cycle (Figure 1; Reiter et al., 2014). Other cells that synthesize melatonin may release the product into the extracellular space but not into the blood. While the pineal gland is touted as the main site of melatonin synthesis, the total amount of melatonin originating from the pineal is small compared to that produced in other cells (Acuña-Castroviejo et al., 2014; Touitou et al., 2017). In the pineal gland, the endogenous rhythm of secretion is regulated by the activity of suprachiasmatic nuclei, the activity of which, in turn, is influenced by the light perception by the retina (Figure 1; Pfeffer et al., 2018). Significant roles of melatonin include conveying circadian information related to the daily cycle of light and darkness and the regulation of immune system function, antioxidant defenses, glucose metabolism, angiogenesis, etc (Manchester et al., 2015; Tordjman et al., 2017; Zhou et al., 2018). The binding of norepinephrine to adrenergic receptors on the pinealocytes is the accepted mechanism leading to the nocturnal synthesis of melatonin by the pineal gland. Following norepinephrine-adrenergic receptor engagement, the activation of adenylate cyclase increases cAMP and *de novo* synthesis of serotonin-N-acetyl transferase, the rate-limiting enzyme in melatonin production (González et al., 2012). In addition to the ligand-receptor pathway, the melatonin synthesis rate may be influenced by intracellular tryptophan and substrate (serotonin) availability and by folic acid and pyridoxine (Zhao D. et al., 2019). Two membrane-bound melatonin receptors (MT1 and MT2) have been identified on pinealocyte membranes which trigger the changes in cAMP through inhibitory G proteins. The increase of cAMP activates downstream effectors like phospholipase-C and protein kinase-C through the MAPK/PI3K/Akt axis. It has been suggested that melatonin, because of its high lipophilicity, directly crosses the cell membrane to enter the blood and cerebrospinal fluid. This capacity facilitates melatonin’s bio-distribution and access to all cells in the organism (Tan et al., 2018).

## Melatonin Signaling Pathway

**FIGURE 3:**
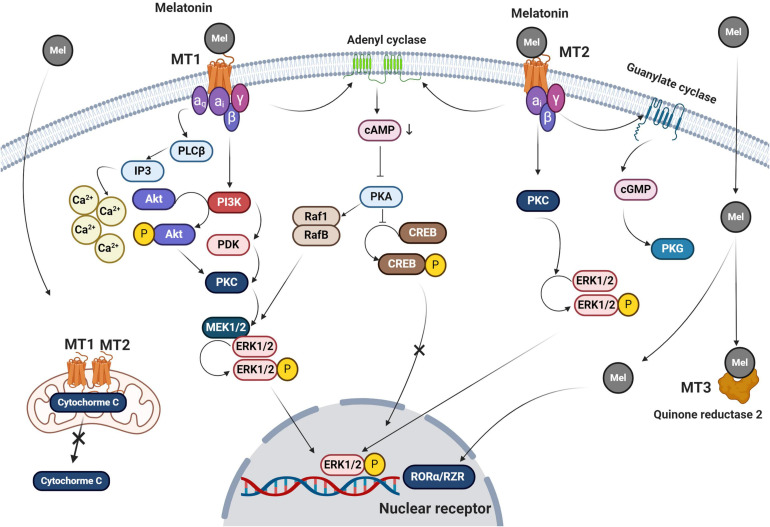
Melatonin signaling pathways are summarized in this figure. Both MT1 and MT2 belong to the GPCRs family of receptors. Melatonin also crosses the plasma membrane via passive diffusion. Upon the attachment of melatonin to MT1 and MT2, different effectors including PKC, PLCβ, and PKA are recruited as second messengers to trigger downstream signaling pathways. For subsequent events, the melatonin-MT1 complex linked α_q_, α_i_, β, and γ subunits to activate IP3 and intracellular accumulation of Ca^+2^ after its release from the endoplasmic reticulum. Ca^+2^ phosphorylates both PKC and ERK1/2. MT1 receptor also acts through membrane adenyl cyclase, which blocks the phosphorylation of CREB by activation of cAMP and PKC. Melatonin binding to MT2 activates the adenyl cyclase and leads to CREB inactivation. Melatonin promotes conformational changes in MT2 and activation of α_*i*_ subunit leads to stimulation of PKG via guanylate cyclase. Also, MT2 engages PKC and ErK1/2 complexes. Melatonin crosses the plasma membrane via passive diffusion and transporters and activates both mitochondrial MT1 and 2 thereby reducing the escape of cytochrome C into the cytosol. Other possible melatonin receptors include cytosolic quinone reductase 2 which activates nuclear RORα/RZR. Mel, melatonin; cAMP, cyclic adenosine monophosphate; cGMP, cyclic guanosine monophosphate; MT, melatonin receptor; PKC, protein kinase C; PKA, protein kinase A; CREB, cAMP response element-binding protein; IP3, inositol trisphosphate; and PDK, pyruvate dehydrogenase kinase.

The melatonin membrane receptors, MT1 and MT2, belong to G-protein coupled receptor (GPCR) subfamily and are widely distributed, accounting for many of melatonin’s pleiotropic functions (Figure 3; Dupré et al., 2018). Besides its receptor-mediated actions, melatonin also enters cells where it has receptor-independent functions (Hardeland, 2009). Once melatonin crosses the plasma membrane, it may also bind to a cytosolic receptor, MT3. The underlying mechanisms supporting the exact role of intracellular MT3 need further investigation.

MT1 and MT2 have seven transmembrane domains and they have several different signaling pathways within cells (Salon et al., 2011). Studies targeting MTs and GPCR indicate that the binding of melatonin to these receptors leads to increased cAMP production, phosphorylation, calcium movements, and morphological adaptation (Ellisdon and Halls, 2016). Results show that MT1 inhibits forskolin-stimulated cAMP formation, phosphorylation of cAMP-responsive element-binding protein (CREB), and PKA activity after melatonin binding (Figure 3; Emet et al., 2016). The activation of MT1 by melatonin is thought to trigger ERK1/2 responsible for cytoskeleton filament remodeling in neuronal cells (Moreno et al., 2020). MT1 and MT2 have glycosylation sites in the extracellular N-terminal region while the cytoplasmic tail consists of fourth intracellular loops and cysteine residues with palmitoylation capability (Dubocovich and Markowska, 2005). The binding of melatonin to MT2 is in accord with phase-shifting and circadian rhythms while MT1 blunts neural firing and phase-shifting (Hunt et al., 2001; Hardeland, 2009). Both MT1 and MT2 are heterodimers and attached to G proteins like α_i__2_, α_i__3_, α_i_, and β and γ. The function of melatonin inside the cells is associated with the activation of diverse subunits of G-proteins connected to MTs and the pattern of downstream interactions.

The activation of MT2 by melatonin is responsible for the reduction of intracellular cAMP ratio and activation of protein kinase C and phospholipase C (Figure 3; Hunt et al., 2001; Rivera-Bermúdez et al., 2004; Birnbaumer, 2007). Activation of G-proteins mediates membrane permeability, allowing ion channels to be opened (Hardeland, 2009). Because of cGMP elevation, it is postulated that this secondary messenger enhances calcium uptake via the cyclic nucleotide-gated channels (Rimler et al., 2007). Melatonin is involved in several transcription processes and gene expression via the regulation of CREB and ERK (Cecon et al., 2018). Binding to multiple intracellular effectors is an additional mechanism-specific to melatonin which is associated with its lipophilic properties (Emet et al., 2016). This is based on the results showing the existence of several putative cytosolic melatonin receptors including enzyme quinone reductase 2 (the MT3 receptor), RORα/RZR nuclear receptors, and calmodulin (Figure 3; Jockers et al., 2008). Evidence points to immune cells as the main sites of RORα1 and RORα2 responsible for mediating melatonin effects while RZRβ is abundant in the pineal gland (Pandi-Perumal et al., 2006). RORα was shown to be an active receptor in the regulation of antioxidant enzymes (Hardeland, 2009). The function of RORα in rat cardiomyocytes after myocardial ischemic reperfusion injury shows its basic role in the control of inflammation and oxidative stress (Figure 3; He et al., 2016). It is similarly observed that melatonin reaches different cellular constituents through its lipophilic property and via receptor-independent pathways (Venegas et al., 2012).

## Effect of Melatonin on Exosome Biogenesis and Release

**FIGURE 4:**
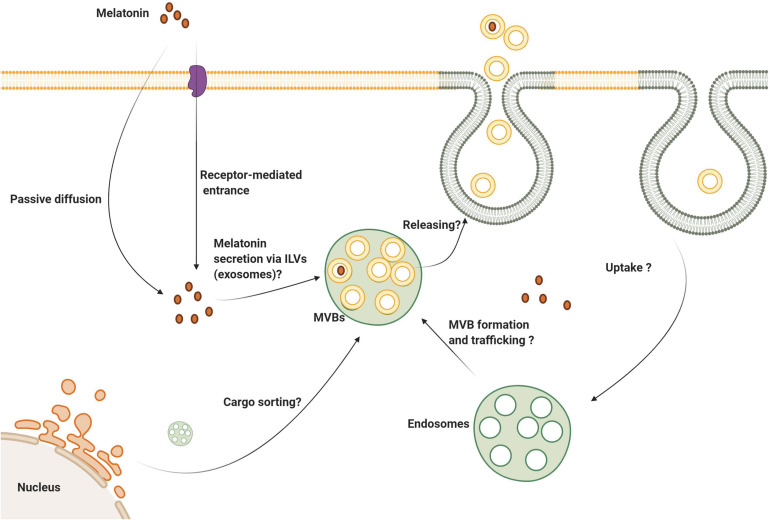
Several aspects of melatonin’s effect on exosome biogenesis and secretion are still unknown. Melatonin can enter the cells via membrane-bound receptors and passive diffusion. It is logical to state that melatonin can be secret via the exosomes to the extracellular niche. Whether melatonin directly or indirectly influences exosome biogenesis, trafficking and abscission need further investigation.

Due to the unique diverse functions of melatonin, it is considered a possible modulator of exosome biogenesis and function (Yoon et al., 2020). How melatonin modulates the function of exosomes under physiological and pathological conditions is under investigation (Figure 4). By identifying the mechanism of action of melatonin as it relates to exosome function, will allow researchers and clinicians to take advantage of the known functions of these important microvesicles. As an example, this may allow for the control of stem cell therapeutic effects in the context of regeneration and other pathological conditions. Using a variety of experimental models in stem cell research, knowledge related to the possible role of exosomes in the alleviation of pathological changes has accumulated (Bang and Kim, 2019; Park et al., 2019; Zhao L. et al., 2019). Recent investigations have established the synergistic effect of melatonin and adipose-derived mesenchymal stem cell (MSC) exosomes in suppressing inflammation, oxidative stress, and apoptosis in a rat model of hepatic ischemia/reperfusion (I/R) (Sun et al., 2017; Chang et al., 2019).

These data are also consistent with other observations showing that exosomes of bone marrow MSCs pre-conditioned with melatonin have benefits in MSCs via paracrine which improved therapeutic efficacy on I/R induced acute renal failure (Alzahrani, 2019; Zahran et al., 2020). However, the exact relevance of melatonin as to its activities, such as those conveyed by exosomes, remains unknown (He et al., 2016). Based on our recent findings melatonin alters exosome size and production in bovine granulosa cells in a dose-dependent manner in which higher melatonin concentrations contribute to the elevated release of small-sized exosomes (Pournaghi et al., 2021). This may be due to the promotion of ILVs formation and/or alterations of exosome physicochemical properties.

**FIGURE 5:**
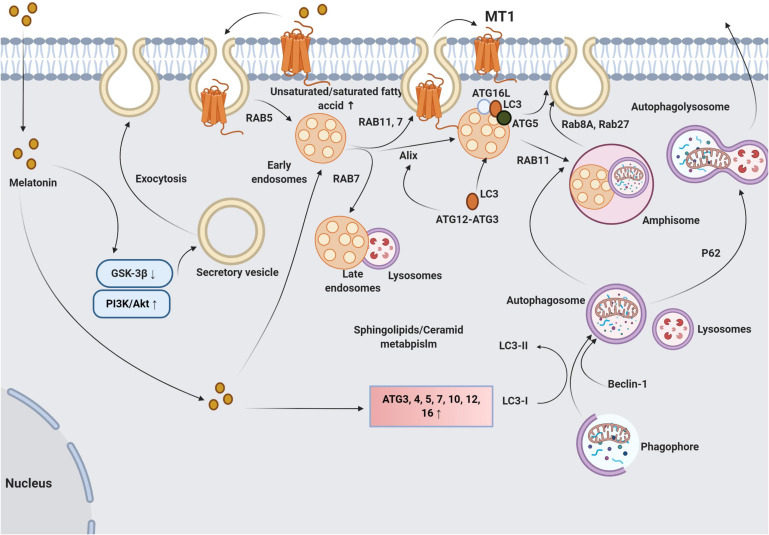
The multiple possible means of molecular cross-talk between melatonin and exosome synthesis machinery are summarized here. Irrespective of melatonin how melatonin enters cells, it activates PI3K/Akt complex and inhibits GSK-3β, leading to fusion of secretory vesicles with plasma membrane and exocytosis. However, the underlying mechanisms have not been fully established. Changes in plasma membrane fluidity by an alteration of fatty acid composition (unsaturated/saturate fatty acid ratio) facilitate the exosome release. Also, melatonin promotes specific GTPases including Rab7 and 11 which accelerate the fusion of late MVBs with the plasma membrane. Upon binding of melatonin to MT1, the melatonin-MT1 complex is transferred to the early endosomes via vacuolar sorting machinery such as Rab5. The activation of Rab7 and 11 promotes endosomal secretion and recycles MT1 to the plasma membrane. Further activation of Rab7 directs endosomes with MT1 toward lysosomes which regulate the innate cell response to melatonin. How Rab 7 determines the fate of endosomes with melatonin (secretion or enzymatic digestion) needs further explorations. The interplay between melatonin and autophagy signaling pathway also influences the activity of exosome molecular machinery. Melatonin stimulates autophagic response directly via the activation of ATG4, 5, 7, 10, 12, and 16 and increases the LC3II/I ratio. These features promote the formation of autophagolysosomes and autophagic exocytosis. Melatonin enhances the autophagic machinery including LC3, ATG3, 5, 12, and 16L on the late endosome membrane that facilitates the fusion of these elements with the plasma membrane. Melatonin also increases the intersection of autophagic vacuoles and exosomes into compartments named amphisomes which have multiple fates. Amphisomes can fuse with lysosomes via the activation of P62. The activity of Rab8 and 27a initiates the autophagic release of amphisomes to the ECM. GSK-3, glycogen synthase kinase 3; ATG, autophagy-related protein; P62, sequestosome 1; LC3, microtubule-associated protein 1A/1B-light chain 3; and PI3K/Akt, Phosphatidylinositol-3-kinase/Protein kinase B.

Whether melatonin is also transferred via exosomes between the cells needs further investigation. The direct passive diffusion of melatonin through cell membranes is accepted. Also, oligopeptide and glucose transporters are alternate routes to deliver melatonin into the cytosol (Venegas et al., 2012; Mayo et al., 2017). Given its lipophilicity and rapid passive diffusion, it is speculated that melatonin transfers horizontally between cells independent of membrane transporters. Interestingly, the transfer of MT1 via internalization, endocytic transport has been explored directly (Abd-Elhafeez et al., 2017). Once melatonin binds MT1, vacuolar sorting machinery transfers the internalized MT1 to the early endosomes using Rab5. Due to the activity of other GTPases like Rab11 and 7, endosomes carrying MT1 can be recycled to the plasma membrane. Alternatively, late endosomes with surface Rab7 activity fuse with the lysosomes (Abd-Elhafeez et al., 2017). The inhibition of β-arrestin-2 by Valproic acid reduces basal endocytic transport of MT1 (Abd-Elhafeez et al., 2017). These data show the potential capacity of exosome transport in intracellular localization of the downstream effectors of the MT signaling pathway (Figure 5).

Although investigated to a lesser extent, the modulation of exosome biogenesis and changes in cargo composition in host cells has been studied. Using an *in vitro* model of Alzheimer’s disease, Ozansoy et al. (2020) showed a reduction of tau carried by exosomes in human neuroblastoma cell line SHSY-5Y incubated with melatonin. They also observed that melatonin pre-treatment of cells before amyloid-β incubation did not affect exosomal tau levels or the intracellular hyperphosphorylated tau content (Ozansoy et al., 2020). A recent histological analysis of sheep testes showed enhanced exosome secretion from cytoplasmic extensions of telocytes located in seminal vesicles after administration of melatonin (Abd-Elhafeez et al., 2017). Following the application of melatonin, the morphology of telocytes was changed and the number and diameter of releasable exosomes increased (Abd-Elhafeez et al., 2017). This issue contrasts with some other results and the difference regarding melatonin’s effect on exosome secretion may be associated with the final concentration of melatonin (either physiological or pharmacological dose). On the basis of the published data, and despite its unique functions in different cell types, it is proposed that melatonin has the potential to regulate exocytosis and exosome delivery.

It is unclear why and how melatonin increases or decreases exosome secretion under different metabolic circumstances. Whether the dose of melatonin or the physiological state of a cell is influential in the exocytosis rate should be further examined. Moreover, the precise signaling pathways involved in exosome biogenesis and secretion upon treatment with melatonin remain unclear. Attempts to identify a correlation between melatonin receptors and exocytosis yielded evidence of relevant signaling cascades (Liu et al., 2015). This group found that the Akt/GSK-3β/CRMP-2 axis is an intermediate molecular process between MT2 and exocytosis in rat hippocampal neurons (Figure 5; Liu et al., 2015). Upon treatment of neurons with melatonin and activation of MT2, the PI3K/Akt axis suppressed GSK-3β activity and reduced phosphorylation of CRMP-2 allowing axonogenesis, exocytosis, and synaptic transmission (Liu et al., 2015). This pathway may be relevant to the regulation of exosome secretion and other extracellular vesicle types.

A quantitative analysis of multiples genes related to different signaling transduction pathways in *Saccharomyces cerevisiae* under oxidative stress revealed mild to moderate expression of 29 genes regulating transmembrane transport activity after treatment with melatonin (Sunyer-Figueres et al., 2020). It may be that whether and how simultaneous activation of MT1 and 2 influence exosome delivery is determined by the physiological state of the host cell. In addition to the molecular pathways that participate in exosome release, other findings indicate changes in the physicochemical properties of the cell membrane as a result of melatonin exposure (Sunyer-Figueres et al., 2020). The report claimed a moderate rise in the synthesis of unsaturated fatty acids and sphingolipids upon treatment with melatonin, indicating an increase in membrane flexibility and fluidity (Sunyer-Figueres et al., 2020). In our previous work, we found that melatonin can alter the unsaturated/saturated fatty acid ratio by increasing Arachidonic acid, Oleic acid, and Linoleic acid levels and change the flexibility of the cell membrane, leading to enhanced exosome delivery (Pournaghi et al., 2021).

This contrasts with the application of long-chain saturated fatty acids like palmitic acid on exosome release (Maly and Hofmann, 2020). It was suggested that the addition of 25 μM palmitic acid suppressed exosome release from prostatic carcinoma PC3 cells (Maly and Hofmann, 2020). The selective inhibition of sphingolipid-metabolizing enzymes such as neutral sphingomyelinase reduced exosome secretion from neural cell lineages (Yuyama et al., 2012). Collectively, the data suggest that an alteration in the metabolism of fatty acids by melatonin may be another molecular mechanism that influences exosome release from host cells.

It is also apparent that melatonin regulates exocytosis which is involved in the progression of apoptosis and autophagy (Luo et al., 2020). Based on the results of several different experiments, certain intracellular and cell-membrane bound proteins are responsible for the fusion and transfer of multiple sets of vesicles. The emerging data point to the existence of subsets of molecular mechanisms shared between autophagy and exosome abscission (Xu et al., 2018). Autophagy-related proteins (ATG), consisting of different subsets, are key regulators of the autophagic response in the cytosol and plasma membrane (Hassanpour et al., 2018a). Data show that ATG5 and ATG16L1 are shared between autophagy signaling and exosome biogenesis (Guo et al., 2017). ATG5 separates vacuolar proton pumps, namely V_1_V_o_-ATPase, and inhibits acidification inside the MVBs, allowing the fusion of MVBs with the plasma membrane and release of ILVs (Figure 5; Xu et al., 2018). The treatment of endothelial progenitor cells with melatonin after exposure to advanced glycation end product decreased cell toxicity by autophagic response mediated by the up-regulation of P-62 and LC3II/I ratio (Jin et al., 2018). It is also suggested once the conjugation of LC3 is initiated via the activity of the ATG3-ATG12 complex, Alix, which belongs to the ESCRT complex, is provoked and interacts with LC3 (Murrow et al., 2015). Thus, cells with LC3 activity achieve the autophagic response and exosome abscission by the interaction of Alix with LC3, showing the integration of autophagy mechanism and exosome biogenesis. In the support of this claim, the inhibition of LC3 conjugation interrupts both exosome secretion and the autophagic response (Murrow et al., 2015). It should be noted, however, that the promotion of autophagy does not necessarily accelerate the secretion of ILVs.

It has been reported that the activation of autophagy-related machinery by rapamycin increased MVB-autophagosome fusion and generated hybrid vacuoles, referred to as amphisomes (Xu et al., 2018). Amphisomes release exosomal and autophagy contents using specific GTPases including Rab8a and Rab27a. Under some circumstances, amphisomes fuse with the cell membrane and are redirected toward lysosomes leading to the formation of autophagolysosomes and a reduction of exosome delivery (Figure 5; Villarroya-Beltri et al., 2016). It was suggested that the expression of GTPases such as Rab27a and Rab27b was increased in cumulus cells after treatment with melatonin for 7 days (Pournaghi et al., 2021).

Studies using endothelial progenitor cells treated with diabetic sera showed reduced exosome secretory capacity (Hassanpour et al., 2018b). Considering the shared machinery regulating exosome abscission and autophagy, the promotion of the autophagic response would be expected to stimulate exosome secretion in the presence of melatonin. However, direct evidence for autophagy-mediated exosome release after melatonin treatment is lacking.

**FIGURE 6:**
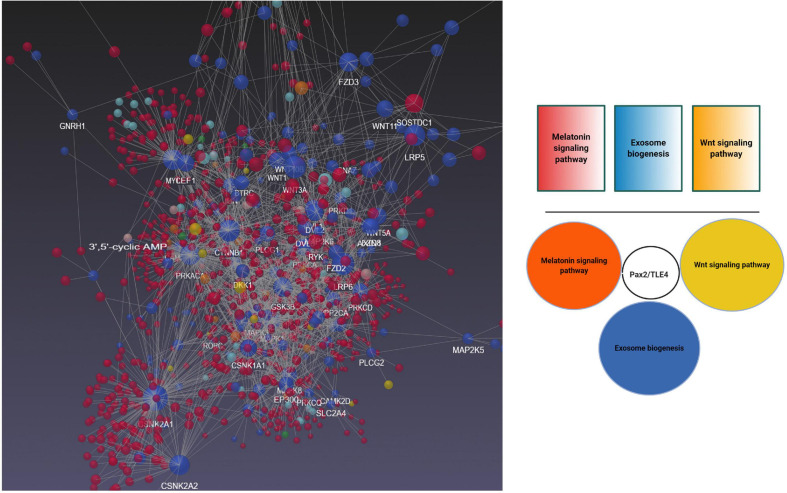
Bioinformatics Analysis of Gene Expression Profiles of Wnt and melatonin signaling pathways with exosome biogenesis using NetworkAnalyst version 3. Data revealed shared Pax2/TLE4 interaction between Wnt, melatonin signaling pathway with exosome biogenesis.

To better understand the possible modulatory effect of melatonin on exosome biogenesis, we performed bioinformatic analyses using NetworkAnalyst version 3, and molecular networks were generated by the application of Network Visual Analytics (Figure 6). Based on our previous experience, the Wnt signaling pathway affects exosome biogenesis and secretion during physiological and pathological conditions of the endothelial lineage (Bagheri et al., 2018). To mine the relationship between melatonin and exosomes, we performed a bioinformatic analysis based on common genes related to the melatonin signaling pathway, exosomes biogenesis, and the Wnt cascade. Our analyses showed that transducin-like enhancer of split 4 (TLE4) is a common effector that cross-links these three pathways. TLE4 belongs to the TLE family of transcription co-repressors. The TLE family members do not directly attach to the DNA. These proteins could generate repressor complexes after binding to Runx2, Hes1, and T cell factor/lymphoid enhancer-binding factor (TCF/LEF) (Zhang et al., 2019). Upon the activation of toll-like receptors (TLRs) including TLR2, 3, 4, and 7 by different ligands, the expression of TLE3 and 4 was initiated under pro-inflammatory status (Zhang et al., 2019). Based on our analyses, we found an interaction of TLE4 with Pax2 shared between melatonin and exosome biogenesis. Previously, the close interaction of melatonin and Wnt signaling pathways has been documented. As a result, the activation of Wnt4 through the ERK1/2-Pax2-Egr1 axis in preosteoblasts after exposure to melatonin increased bone formation capacity (Li et al., 2019). Whether activation of Pax2/TLE4 complex modulates the biogenesis of exosomes needs further investigations.

Research advances have confirmed the role of melatonin as a key player in the regulation of cell paracrine responses via exosome biogenesis. Further investigations should be directed at the characterization of the signaling pathways that regulate exosome biogenesis after treatment with melatonin. Identifying the molecular events by which melatonin modulates exosome biogenesis will likely soon become an active area of investigation.

## Effect of Melatonin on Exosome Cargo Sorting

Within the last decade, *in vivo* and *in vitro* evidence has shown that melatonin changes the molecular composition of exosome cargo (Table 1; Ozansoy et al., 2020). Progressive chronic changes such as chronic kidney disease, profoundly decrease the innate restorative capacity of MSCs (Zhao et al., 2020). Moreover, molecular analyses confirmed the existence of several factors, in the particular cellular prion protein (PrPC) and miR-4516, which participate in the regulation of angiogenesis (Alzahrani, 2019). These changes occurred concurrently with other desirable effects such as anti-aging actions and improvement of mitochondrial function after transplantation of MSCs treated with melatonin. These studies revealed that melatonin stimulates MSCs to release exosomes that are highly enriched with miR-4516-PrPC (Yoon et al., 2020). Other pro-angiogenic and anti-inflammatory factors have also been identified in MSC-derived exosomes pre-conditioned with melatonin (Liu et al., 2020). Exosomes isolated from melatonin-treated MSCs accelerated healing via an increase of microvascular density and suppression of inflammation in the rat model of diabetic wound healing (Liu et al., 2020).

**TABLE 1:** Melatonin could alter exosomal cargo.

**Type of study**	**Study design**	**Outcome**	**References**
*In vivo* and *in vitro*	• Injection of bone marrow MSC-derived exosomes exposed to melatonin in a rat model of diabetic wound healing	IL-1β↓, TNF-α↓, and IL-10↑, Arg-1↓ and iNOS↓, Improved angiogenesis rate (CD31 and α-SMA↑), collagen synthesis↑, Increased M2 to M1 polarization via activation of PTEN/AKT signaling pathway	Liu et al., 2020
*In vivo*	• Hepatoprotection of adipocyte-derived exosomes in mice received melatonin compared to mice fed with a high-fat diet and resistin	Fatty acid accumulation in the liver↓, endoplasmic reticulum stress↓, hepatic steatosis↓, resistin-induced AMPKα phosphorylation↓, increased resistin mRNA degradation↑, Bmal1 transcriptional inhibition↑, and m^6^ A RNA demethylation in adipocytes ↑	Rong et al., 2019
*In vitro and in vivo*	• Melatonin-treated hepatic carcinoma-exosomes effect on peritoneal macrophages immunosuppression capacity	Peritoneal macrophage phosphorylated STAT3↓, IL-6↓, IL-1β↓, IL-10↓, PD-L1↓, and TNF-α↓ in THP-1 cells,	Cheng et al., 2017
*In vivo*	• Maternal intraperitoneal of melatonin on lipopolysaccharide-induced neonatal brain inflammation	Microglial activity↓, Endoplasmic reticulum↓, Autophagy efflux↑, eIF2α phosphorylation↑, P62 and LC3↑, SIRT1↑, miR-34a↓, miR146a↓, and miR-126↓, Inflammatory response↓	Carloni et al., 2016
*In vivo*	• Injection of MSCs exosomes preconditioned with melatonin in a rat model of renal I/R injury	Tubular epithelial cell necrosis↓, Immune cell infiltration, and inflammation↓, BUN and creatinine levels ↓, Renal BUN and creatinine levels↑, MDA↓, NOx↓, HIF-1α↑, HO1↑, Apoptosis-related markers↓, Angiogenesis↑	Alzahrani, 2019

*↓ Decrease; ↓ Increase; IL-1β, interleukin 1 beta; TNFα, tumor necrosis factor α; ARG1, arginase; iNOS, inducible nitric oxide synthase; αSMA, smooth muscle alpha-actin; CD31, platelet endothelial cell adhesion molecule; AMPKα, AMP-activated protein kinase α subunit; P62, sequestosome-1; LC3, microtubule-associated protein 1A/1B-light chain 3; PD-L1, programmed death-ligand 1; STAT3, signal transducer and activator of transcription 3; eIF2, eukaryotic initiation factor 2; BUN, blood urea nitrogen; MDA, malondialdehyde; NOx, nitrogen oxides; HIF-1α, hypoxia-inducible factor 1 alpha; HO-1, heme oxygenase-1.*

Evidence for therapeutic effects of exosomes in hepatic disease has also been noted (Shen et al., 2014; Colica and Abenavoli, 2018). A recent study explored the hepato-protection of adipocyte-derived exosomes in mice who received melatonin (Rong et al., 2019). Compared to mice fed with resistin and a high-fat diet, melatonin application produced adipocyte-derived exosomes which are capable of reducing hepatic steatosis and endoplasmic reticulum stress (Rong et al., 2019). These data suggest that melatonin changes the exosome cargo and decreases phosphorylation of AMPK-α in Thr172 residue and that this activity prohibits endoplasmic reticulum and the development of hepatic steatosis (Rong et al., 2019). Owing to melatonin’s pleiotropic effects, one scenario is that melatonin displays a regulatory mechanism on small molecules known as miRNAs which are subsequently released by exosomes (Hardeland, 2018a). Melatonin appears to alter m6 A RNA demethylation in adipocytes and changes the expression of resistin (Rong et al., 2019).

A large number of cancer cell exosomes, known also as oncosomes, containing multiple sets of miRNAs are involved in tumor metastasis and expansion (Cufaro et al., 2019; Jaiswal and Sedger, 2019). Melatonin is known to regulate the transcription of certain miRNAs such as miR-155 and miR-21 in cancer cells. These miRNAs are responsible for tumor growth and metastasis (Hardeland, 2018b; Hunsaker et al., 2019). The alteration of exosomal miRNAs by melatonin not only inhibits the horizontal transfer of anaplastic-associated bio-information by exosomes but also recruits immune cells to the cancer site (Alfonsi et al., 2018; Wang et al., 2019). Studies have shown a moderate reduction in pro-inflammatory cytokines including TNF-α, IL-6, -10, -1β and programmed death-ligand 1 by macrophages incubated with hepatocellular carcinoma-derived exosomes pre-conditioned with melatonin (Cheng et al., 2017). The regulation of STAT3 may play a critical role in the immunomodulatory actions of carcinoma-derived exosomes (Cheng et al., 2017). Under other circumstances, such as lipopolysaccharide-induced Alzheimer’s disease in rat neonates, melatonin decreases the pathological complications via the autophagy-SIRT-1 pathway and down-regulation of multiple miRNAs including miR-34a, -146a, and -126 (Meng et al., 2014; Carloni et al., 2016). Similarly, Heo et al. (2020) showed that melatonin-stimulated exosomes isolated from adipose-derived mesenchymal stem cells suppressed inflammation. These exosomes were enriched with multiple miRNAs such as miR-34a, -124, and -135b compared to non-treated melatonin exosomes.

Since various accessory factors are shared by autophagy and exosome signaling pathways, it is presumed that melatonin inhibits the progression of Alzheimer’s disease by activation of exocytosis via both autophagy efflux and exosome secretion. This possibility as it relates to neurodegenerative diseases needs further analysis.

A recent study reported by Wang et al. (2020) injected blood-derived exosomes of melatonin-treated rats into the brain after focal cerebral ischemia. Melatonin changed the exosomal miRNAs profile and reduced ischemic changes via the promotion of angiogenesis, neurogenesis, and suppression of apoptosis. It is felt that melatonin alters cargo sorting in the exosomes. Attempts to investigate the effect of melatonin on exosome functions have not yielded a definition of the molecular mechanisms by which it controls exosome dynamics.

## Concluding Remarks and Perspectives

Research progress has led to the partial clarification of melatonin’s efficacy in the modulation of exosomal biogenesis. What has been uncovered is that melatonin can alter the exosome biogenesis, intracellular trafficking, and abscission at the molecular levels by engaging different effectors. Further investigations should be focused on the identification of commonly shared pathways between exosome biogenesis and the melatonin signaling cascade. Based on the dose- and context-dependent activity of melatonin on the target cell populations, the findings related to these interactions will have to be interpreted with caution. Questions related to the heterogeneity and functions of effectors shared between two signaling pathways are still unanswered. Advancements in molecular biological analyses and improved technologies will be aids in clarifying melatonin-exosome interactions at different subcellular levels.

## Author Contributions

HA, AR, MHe, MHa, SH, and ES collected the data and prepared the draft. SG performed the bioinformatic analysis. RR and RJR edited and supervised the manuscript. All authors contributed to the article and approved the submitted version.

## Conflict of Interest

The authors declare that the research was conducted in the absence of any commercial or financial relationships that could be construed as a potential conflict of interest.

## References

[B1] Abd-Elhafeez H. H. Mokhtar D. M. Hassan A. H. (2017). Effect of melatonin on telocytes in the seminal vesicle of the Soay ram: an immunohistochemical, ultrastructural and morphometrical study. *Cells Tissues Organs* 203 29–54. 10.1159/000449500 27802428

[B2] Acuña-Castroviejo D. Escames G. Venegas C. Diaz-Casado M. E. Lima-Cabello E. López L. C. (2014). Extrapineal melatonin: sources, regulation, and potential functions. *Cell. Mol. Life Sci.* 71 2997–3025. 10.1007/s00018-014-1579-2 24554058 PMC11113552

[B3] Ageta H. Tsuchida K. (2019). Post-translational modification and protein sorting to small extracellular vesicles including exosomes by ubiquitin and UBLs. *Cel. Mol. Life Sci.* 76 4829–4848. 10.1007/s00018-019-03246-7 31363817 PMC11105257

[B4] Alfonsi R. Grassi L. Signore M. Bonci D. (2018). The double face of exosome-carried microRNAs in cancer immunomodulation. *Int. J. Mol. Sci.* 19:1183. 10.3390/ijms19041183 29652798 PMC5979514

[B5] Alzahrani F. A. (2019). Melatonin improves therapeutic potential of mesenchymal stem cells-derived exosomes against renal ischemia-reperfusion injury in rats. *Am. J. Transl. Res.* 11 2887–2907. 31217862 PMC6556638

[B6] Bagheri H. S. Mousavi M. Rezabakhsh A. Rezaie J. Rasta S. H. Nourazarian A. (2018). Low-level laser irradiation at a high power intensity increased human endothelial cell exosome secretion via Wnt signaling. *Lasers Med. Sci.* 33 1131–1145. 10.1007/s10103-018-2495-8 29603107

[B7] Bang O. Y. Kim E. H. (2019). Mesenchymal stem cell-derived extracellular vesicle therapy for stroke: challenges and progress. *Front. Neurol.* 10:211. 10.3389/fneur.2019.00211 30915025 PMC6422999

[B8] Birnbaumer L. (2007). Expansion of signal transduction by G proteins: the second 15 years or so: from 3 to 16 α subunits plus βγ dimers. *Biochim. Biophys. Acta* 1768 772–793. 17258171 10.1016/j.bbamem.2006.12.002 PMC1993906

[B9] Carloni S. Favrais G. Saliba E. Albertini M. C. Chalon S. Longini M. (2016). Melatonin modulates neonatal brain inflammation through endoplasmic reticulum stress, autophagy, and miR-34a/silent information regulator 1 pathway. *J. Pineal Res.* 61 370–380. 10.1111/jpi.12354 27441728

[B10] Castillo R. R. Quizon G. R. A. Juco M. J. M. Roman A. D. E. de Leon D. G. Punzalan F. E. R. (2020). Melatonin as adjuvant treatment for coronavirus disease 2019 pneumonia patients requiring hospitalization (MAC-19 PRO): a case series. *Melatonin Res.* 3 297–310. 10.32794/mr11250063

[B11] Cecon E. Oishi A. Jockers R. (2018). Melatonin receptors: molecular pharmacology and signalling in the context of system bias. *Br. J. Pharmacol.* 175 3263–3280. 10.1111/bph.13950 28707298 PMC6057902

[B12] Chang C.-L. Chen C.-H. Chiang J. Y. Sun C.-K. Chen Y.-L. Chen K.-H. (2019). Synergistic effect of combined melatonin and adipose-derived mesenchymal stem cell (ADMSC)-derived exosomes on amelioration of dextran sulfate sodium (DSS)-induced acute colitis. *Am. J. Transl. Res.* 11 2706–2424. 31217848 PMC6556660

[B13] Cheng L. Liu J. Liu Q. Liu Y. Fan L. Wang F. (2017). Exosomes from melatonin treated hepatocellularcarcinoma cells alter the immunosupression status through STAT3 pathway in macrophages. *Int. J. Biol. Sci.* 13 723–734. 10.7150/ijbs.19642 28655998 PMC5485628

[B14] Claustrat B. Leston J. (2015). Melatonin: physiological effects in humans. *Neurochirurgie* 61 77–84. 10.1016/j.neuchi.2015.03.002 25908646

[B15] Colica C. Abenavoli L. (2018). Resistin levels in non-alcoholic fatty liver disease pathogenesis. *J. Transl. Int. Med.* 6 52–53. 10.2478/jtim-2018-0011 29607306 PMC5874489

[B16] Colombo M. Moita C. van Niel G. Kowal J. Vigneron J. Benaroch P. (2013). Analysis of ESCRT functions in exosome biogenesis, composition and secretion highlights the heterogeneity of extracellular vesicles. *J. Cell Sci.* 126 5553–5565. 10.1242/jcs.128868 24105262

[B17] Colombo M. Raposo G. Théry C. (2014). Biogenesis, secretion, and intercellular interactions of exosomes and other extracellular vesicles. *Annu. Rev. Cell Dev. Biol.* 30 255–289. 10.1146/annurev-cellbio-101512-122326 25288114

[B18] Cufaro M. C. Pieragostino D. Lanuti P. Rossi C. Cicalini I. Federici L. (2019). Extracellular vesicles and their potential use in monitoring cancer progression and therapy: the contribution of proteomics. *J. Oncol.* 2019:1639854. 31281356 10.1155/2019/1639854 PMC6590542

[B19] Dubocovich M. L. Markowska M. (2005). Functional MT 1 and MT 2 melatonin receptors in mammals. *Endocrine* 27 101–110. 10.1385/endo:27:2:101 16217123

[B20] Dupré C. Bruno O. Bonnaud A. Giganti A. Nosjean O. Legros C. (2018). Assessments of cellular melatonin receptor signaling pathways: β-arrestin recruitment, receptor internalization, and impedance variations. *Eur. J. Pharmacol.* 818 534–544. 10.1016/j.ejphar.2017.11.022 29154938

[B21] Ellisdon A. M. Halls M. L. (2016). Compartmentalization of GPCR signalling controls unique cellular responses. *Biochem. Soc. Trans.* 44 562–567. 10.1042/bst20150236 27068970

[B22] Emet M. Ozcan H. Ozel L. Yayla M. Halici Z. Hacimuftuoglu A. (2016). A review of melatonin, its receptors and drugs. *Eurasian J. Med.* 48 135–141. 10.5152/eurasianjmed.2015.0267 27551178 PMC4970552

[B23] Fitzner D. Schnaars M. van Rossum D. Krishnamoorthy G. Dibaj P. Bakhti M. (2011). Selective transfer of exosomes from oligodendrocytes to microglia by macropinocytosis. *J. Cell Sci.* 124 447–458. 10.1242/jcs.074088 21242314

[B24] González S. Moreno-Delgado D. Moreno E. Pérez-Capote K. Franco R. Mallol J. (2012). Circadian-related heteromerization of adrenergic and dopamine D4 receptors modulates melatonin synthesis and release in the pineal gland. *PLoS Biol.* 10:e1001347. 10.1371/journal.pbio.1001347 22723743 PMC3378626

[B25] Guo H. Chitiprolu M. Roncevic L. Javalet C. Hemming F. J. Trung M. T. (2017). Atg5 disassociates the V_1_V_0_-ATPase to promote exosome production and tumor metastasis independent of canonical macroautophagy. *Dev. Cell* 43 716.e7–730.e7. 29257951 10.1016/j.devcel.2017.11.018

[B26] Hardeland R. (2009). Melatonin: signaling mechanisms of a pleiotropic agent. *Biofactors* 35 183–192. 10.1002/biof.23 19449447

[B27] Hardeland R. (2018a). Extended signaling by melatonin. *Cell Cell Life Sci J.* 3:000123.

[B28] Hardeland R. (2018b). Interactions of melatonin and microRNAs. *Biochem. Mol. Biol. J.* 4:7. 10.21767/2471-8084.100056

[B29] Hassanpour M. Rezabakhsh A. Pezeshkian M. Rahbarghazi R. Nouri M. (2018a). Distinct role of autophagy on angiogenesis: highlights on the effect of autophagy in endothelial lineage and progenitor cells. *Stem Cell Res. Ther.* 9:305. 10.1016/b978-0-12-405877-4.00021-4 30409213 PMC6225658

[B30] Hassanpour M. Cheraghi O. Brazvan B. Hiradfar A. Aghamohammadzadeh N. Rahbarghazi R. (2018b). Chronic exposure of human endothelial progenitor cells to diabetic condition abolished the regulated kinetics activity of exosomes. *Iranian J. Pharm. Res.* 17 1068–1080. PMC6094433 30127829

[B31] He B. Zhao Y. Xu L. Gao L. Su Y. Lin N. (2016). The nuclear melatonin receptor ROR α is a novel endogenous defender against myocardial ischemia/reperfusion injury. *J. Pineal Res.* 60 313–326. 10.1111/jpi.12312 26797926

[B32] Heo J. S. Lim J.-Y. Yoon D. W. Pyo S. Kim J. (2020). Exosome and Melatonin Additively Attenuates Inflammation by Transferring miR-34a, miR-124, and miR-135b. *Biomed Res. Int.* 2020:1621394. 33299858 10.1155/2020/1621394 PMC7707940

[B33] Hessvik N. P. Llorente A. (2018). Current knowledge on exosome biogenesis and release. *Cell. Mol. Life Sci.* 75 193–208. 10.1007/s00018-017-2595-9 28733901 PMC5756260

[B34] Hooper C. Sainz-Fuertes R. Lynham S. Hye A. Killick R. Warley A. (2020). Correction to: Wnt3a induces exosome secretion from primary cultured rat microglia. *BMC Neurosci.* 21:10. 10.1186/s12868-020-0558-9 32138650 PMC7059358

[B35] Huang-Doran I. Zhang C.-Y. Vidal-Puig A. (2017). Extracellular vesicles: novel mediators of cell communication in metabolic disease. *Trends Endocrinol. Metab.* 28 3–18. 10.1016/j.tem.2016.10.003 27810172

[B36] Hunsaker M. Barba G. Kingsley K. Howard K. M. (2019). Differential microRNA expression of miR-21 and miR-155 within oral cancer extracellular vesicles in response to melatonin. *Dent. J.* 7 48. 10.3390/dj7020048 31052365 PMC6631699

[B37] Hunt A. E. Al-Ghoul W. M. Gillette M. U. Dubocovich M. L. (2001). Activation of MT2 melatonin receptors in rat suprachiasmatic nucleus phase advances the circadian clock. *Am. J. Physiol. Cell Physiol.* 280 C110–C118. 11121382 10.1152/ajpcell.2001.280.1.C110

[B38] Hurley J. H. (2015). ESCRTs are everywhere. *EMBO J.* 34 2398–2407. 26311197 10.15252/embj.201592484 PMC4601661

[B39] Jaiswal R. Sedger L. M. (2019). Intercellular vesicular transfer by exosomes, microparticles and oncosomes-implications for cancer biology and treatments. *Front. Oncol.* 9:125. 10.3389/fonc.2019.00125 30895170 PMC6414436

[B40] Jin H. Zhang Z. Wang C. Tang Q. Wang J. Bai X. (2018). Melatonin protects endothelial progenitor cells against AGE-induced apoptosis via autophagy flux stimulation and promotes wound healing in diabetic mice. *Exp. Mol. Med.* 50 1–15. 10.1038/s12276-018-0177-z 30459300 PMC6249246

[B41] Jockers R. Maurice P. Boutin J. Delagrange P. (2008). Melatonin receptors, heterodimerization, signal transduction and binding sites: what’s new? *Br. J. Pharmacol.* 154 1182–1195. 10.1038/bjp.2008.184 18493248 PMC2483381

[B42] Kamerkar S. LeBleu V. S. Sugimoto H. Yang S. Ruivo C. F. Melo S. A. (2017). Exosomes facilitate therapeutic targeting of oncogenic KRAS in pancreatic cancer. *Nature* 546 498–503. 10.1038/nature22341 28607485 PMC5538883

[B43] Li X. Li Z. Wang J. Li Z. Cui H. Dai G. (2019). Wnt4 signaling mediates protective effects of melatonin on new bone formation in an inflammatory environment. *FASEB J.* 33 10126–10139. 10.1096/fj.201900093rr 31216173

[B44] Liu D. Wei N. Man H. Y. Lu Y. Zhu L. Q. Wang J. Z. (2015). The MT2 receptor stimulates axonogenesis and enhances synaptic transmission by activating Akt signaling. *Cell Death Differ.* 22 583–596. 10.1038/cdd.2014.195 25501601 PMC4356342

[B45] Liu W. Yu M. Xie D. Wang L. Ye C. Zhu Q. (2020). Melatonin-stimulated MSC-derived exosomes improve diabetic wound healing through regulating macrophage M1 and M2 polarization by targeting the PTEN/AKT pathway. *Stem Cell Res. Ther.* 11:296. 32600435 10.1186/s13287-020-01756-x PMC7322868

[B46] Luo F. Sandhu A. F. Rungratanawanich W. Williams G. E. Akbar M. Zhou S. (2020). Melatonin and autophagy in aging-related neurodegenerative diseases. *Int. J. Mol. Sci.* 21:7174. 10.3390/ijms21197174 32998479 PMC7584015

[B47] Maly I. V. Hofmann W. A. (2020). Effect of palmitic acid on exosome-mediated secretion and invasive motility in prostate cancer cells. *Molecules* 25:2722. 10.3390/molecules25122722 32545453 PMC7355791

[B48] Manchester L. C. Coto-Montes A. Boga J. A. Andersen L. P. H. Zhou Z. Galano A. (2015). Melatonin: an ancient molecule that makes oxygen metabolically tolerable. *J. Pineal Res.* 59 403–419. 10.1111/jpi.12267 26272235

[B49] Matar E. McCarter S. J. St Louis E. K. Lewis S. J. G. (2021). Current concepts and controversies in the management of REM sleep behavior disorder. *Neurotherapeutics* 10.1007/s13311-020-00983-7 Online ahead of print. 33410105 PMC8116413

[B50] Mayo J. C. Sainz R. M. González-Menéndez P. Hevia D. Cernuda-Cernuda R. (2017). Melatonin transport into mitochondria. *Cell. Mol. Life Sci.* 74 3927–3940. 10.1007/s00018-017-2616-8 28828619 PMC11107582

[B51] Meng F. Dai E. Yu X. Zhang Y. Chen X. Liu X. (2014). Constructing and characterizing a bioactive small molecule and microRNA association network for Alzheimer’s disease. *J. R. Soc. Interface* 11:20131057. 10.1098/rsif.2013.1057 24352679 PMC3899875

[B52] Minciacchi V. R. Freeman M. R. Di Vizio D. (2015). Extracellular vesicles in cancer: exosomes, microvesicles and the emerging role of large oncosomesed. *Semin. Cell Dev. Biol.* 40 41–51. 10.1016/j.semcdb.2015.02.010 25721812 PMC4747631

[B53] Moreno A. C. R. de Freitas Saito R. Tiago M. Massaro R. R. Pagni R. L. Pegoraro R. (2020). Melatonin inhibits human melanoma cells proliferation and invasion via cell cycle arrest and cytoskeleton remodeling. *Melatonin Res.* 3 194–209. 10.32794/mr11250057

[B54] Mulcahy L. A. Pink R. C. Carter D. R. F. (2014). Routes and mechanisms of extracellular vesicle uptake. *J. Extracell. Vesicles* 3:24641. 10.3402/jev.v3.24641 25143819 PMC4122821

[B55] Murrow L. Malhotra R. Debnath J. (2015). ATG12–ATG3 interacts with Alix to promote basal autophagic flux and late endosome function. *Nat. Cell Biol.* 17 300–310. 10.1038/ncb3112 25686249 PMC4344874

[B56] Ozansoy M. Ozansoy M. B. Yulug B. Cankaya S. Kilic E. Goktekin S. (2020). Melatonin affects the release of exosomes and tau-content in in vitro amyloid-beta toxicity model. *J. Clin. Neurosci.* 73 237–244. 10.1016/j.jocn.2019.11.046 32061493

[B57] Pandi-Perumal S. R. Srinivasan V. Maestroni G. Cardinali D. Poeggeler B. Hardeland R. (2006). Melatonin: nature’s most versatile biological signal? *FEBS J.* 273 2813–2838. 10.1111/j.1742-4658.2006.05322.x 16817850

[B58] Park K.-S. Bandeira E. Shelke G. V. Lässer C. Lötvall J. (2019). Enhancement of therapeutic potential of mesenchymal stem cell-derived extracellular vesicles. *Stem Cell Res. Ther.* 10:288. 31547882 10.1186/s13287-019-1398-3 PMC6757418

[B59] Pfeffer M. Korf H.-W. Wicht H. (2018). Synchronizing effects of melatonin on diurnal and circadian rhythms. *Gen. Comp. Endocrinol.* 258 215–221. 10.1016/j.ygcen.2017.05.013 28533170

[B60] Pournaghi M. Khodavirdilou R. Saadatlou M. A. E. Nasimi F. S. Yousefi S. Mobarak H. (2021). Effect of melatonin on exosomal dynamics in bovine cumulus cells. *Process Biochem.* 106 78–87. 10.1016/j.procbio.2021.03.008

[B61] Record M. (2014). Intercellular communication by exosomes in placenta: a possible role in cell fusion? *Placenta* 35 297–302. 10.1016/j.placenta.2014.02.009 24661568

[B62] Reiter R. J. Ma Q. Sharma R. (2020). Melatonin in mitochondria: mitigating clear and present dangers. *Physiology* 35 86–95. 10.1152/physiol.00034.2019 32024428

[B63] Reiter R. J. Tan D. X. Kim S. J. Cruz M. H. C. (2014). Delivery of pineal melatonin to the brain and SCN: role of canaliculi, cerebrospinal fluid, tanycytes and Virchow–Robin perivascular spaces. *Brain Struct. Funct.* 219 1873–1887. 10.1007/s00429-014-0719-7 24553808

[B64] Rezaie J. Ajezi S. Avci ÇB. Karimipour M. Geranmayeh M. H. Nourazarian A. (2018). Exosomes and their application in biomedical field: difficulties and advantages. *Mol. Neurobiol.* 55 3372–3393. 10.1007/s12035-017-0582-7 28497202

[B65] Rimler A. Jockers R. Lupowitz Z. Zisapel N. (2007). Gi and RGS proteins provide biochemical control of androgen receptor nuclear exclusion. *J. Mol. Neurosci.* 31 1–12. 10.1007/bf02686113 17416965

[B66] Rivera-Bermúdez M. A. Masana M. I. Brown G. M. Earnest D. J. Dubocovich M. L. (2004). Immortalized cells from the rat suprachiasmatic nucleus express functional melatonin receptors. *Brain Res.* 1002 21–27. 10.1016/j.brainres.2003.12.008 14988029

[B67] Rong B. Feng R. Liu C. Wu Q. Sun C. (2019). Reduced delivery of epididymal adipocyte-derived exosomal resistin is essential for melatonin ameliorating hepatic steatosis in mice. *J. Pineal Res.* 66:e12561. 10.1111/jpi.12561 30659651

[B68] Sahu R. Kaushik S. Clement C. C. Cannizzo E. S. Scharf B. Follenzi A. (2011). Microautophagy of cytosolic proteins by late endosomes. *Dev. Cell* 20 131–139. 10.1016/j.devcel.2010.12.003 21238931 PMC3025279

[B69] Salon J. A. Lodowski D. T. Palczewski K. (2011). The significance of G protein-coupled receptor crystallography for drug discovery. *Pharmacol. Rev.* 63 901–937. 10.1124/pr.110.003350 21969326 PMC3186081

[B70] Segura E. Nicco C. Lombard B. Véron P. Raposo G. Batteux F. (2005). ICAM-1 on exosomes from mature dendritic cells is critical for efficient naive T-cell priming. *Blood* 106 216–223. 10.1182/blood-2005-01-0220 15790784

[B71] Shen C. Zhao C.-Y. Wang W. Wang Y.-D. Sun H. Cao W. (2014). The relationship between hepatic resistin overexpression and inflammation in patients with nonalcoholic steatohepatitis. *BMC Gastroenterol.* 14:39. 24559185 10.1186/1471-230X-14-39 PMC3942781

[B72] Soekmadji C. Riches J. D. Russell P. J. Ruelcke J. E. McPherson S. Wang C. (2017). Modulation of paracrine signaling by CD9 positive small extracellular vesicles mediates cellular growth of androgen deprived prostate cancer. *Oncotarget.* 8 52237–52255. 10.18632/oncotarget.11111 28881726 PMC5581025

[B73] Sun C.-K. Chen C.-H. Chang C.-L. Chiang H.-J. Sung P.-H. Chen K.-H. (2017). Melatonin treatment enhances therapeutic effects of exosomes against acute liver ischemia-reperfusion injury. *Am. J. Transl. Res.* 9 1543–1560. 28469765 PMC5411908

[B74] Sunyer-Figueres M. Vázquez J. Mas A. Torija M. J. Beltran G. (2020). Transcriptomic Insights into the effect of melatonin in saccharomyces cerevisiae in the presence and absence of oxidative stress. *Antioxidants* 9 583–596. 10.3390/antiox9100947 PMC7650831 33019712

[B75] Tan D. Xu B. Zhou X. Reiter R. (2018). Pineal calcification, melatonin production, aging, associated health consequences and rejuvenation of the pineal gland. *Molecules* 23:301. 10.3390/molecules23020301 29385085 PMC6017004

[B76] Tan D. X. Manchester L. C. Liu X. Rosales-Corral S. A. Acuna-Castroviejo D. Reiter R. J. (2013). Mitochondria and chloroplasts as the original sites of melatonin synthesis: a hypothesis related to melatonin’s primary function and evolution in eukaryotes. *J. Pineal Res.* 54 127–138. 10.1111/jpi.12026 23137057

[B77] Théry C. Ostrowski M. Segura E. (2009). Membrane vesicles as conveyors of immune responses. *Nat. Rev. Immunol.* 9 581–593. 10.1038/nri2567 19498381

[B78] Tordjman S. Chokron S. Delorme R. Charrier A. Bellissant E. Jaafari N. (2017). Melatonin: pharmacology, functions and therapeutic benefits. *Curr. Neuropharmacol.* 15 434–443. 10.2174/1570159x14666161228122115 28503116 PMC5405617

[B79] Touitou Y. Reinberg A. Touitou D. (2017). Association between light at night, melatonin secretion, sleep deprivation, and the internal clock: health impacts and mechanisms of circadian disruption. *Life Sci.* 173 94–106. 10.1016/j.lfs.2017.02.008 28214594

[B80] Valadi H. Ekström K. Bossios A. Sjöstrand M. Lee J. J. Lötvall J. O. (2007). Exosome-mediated transfer of mRNAs and microRNAs is a novel mechanism of genetic exchange between cells. *Nat. Cell Biol.* 9 654–659. 10.1038/ncb1596 17486113

[B81] Venegas C. García J. A. Escames G. Ortiz F. López A. Doerrier C. (2012). Extrapineal melatonin: analysis of its subcellular distribution and daily fluctuations. *J. Pineal Res.* 52 217–227. 10.1111/j.1600-079x.2011.00931.x 21884551

[B82] Villarroya-Beltri C. Baixauli F. Mittelbrunn M. Fernández-Delgado I. Torralba D. Moreno-Gonzalo O. (2016). ISGylation controls exosome secretion by promoting lysosomal degradation of MVB proteins. *Nat. Commun.* 7:13588. 27882925 10.1038/ncomms13588 PMC5123068

[B83] Wang K. Ru J. Zhang H. Chen J. Lin X. Lin Z. (2020). Melatonin enhances the therapeutic effect of plasma exosomes against cerebral ischemia-induced pyroptosis through the TLR4/NF-κB pathway. *Front. Neurosci.* 14:848. 10.3389/fnins.2020.00848 33013286 PMC7461850

[B84] Wang T. Nasser M. I. Shen J. Qu S. He Q. Zhao M. (2019). Functions of Exosomes in the Triangular Relationship between the Tumor, Inflammation, and Immunity in the Tumor Microenvironment. *J. Immunol. Res.* 2019:4197829. 31467934 10.1155/2019/4197829 PMC6701352

[B85] Wei Y. Bai Y. Cheng X. Zhu B. Reiter R. J. Shi H. (2020). The dual roles of melatonin biosynthesis enzymes in the coordination of melatonin biosynthesis and autophagy in cassava. *J. Pineal Res.* 69:e12652. 32201970 10.1111/jpi.12652

[B86] Whitham M. Parker B. L. Friedrichsen M. Hingst J. R. Hjorth M. Hughes W. E. (2018). Extracellular vesicles provide a means for tissue crosstalk during exercise. *Cell metab.* 27 237.–251. 29320704 10.1016/j.cmet.2017.12.001

[B87] Xu J. Camfield R. Gorski S. M. (2018). The interplay between exosomes and autophagy – partners in crime. *J. Cell Sci.* 131:jcs215210. 30076239 10.1242/jcs.215210

[B88] Yáñez-Mó M. Siljander P. R.-M. Andreu Z. Bedina Zavec A. Borràs F. E. Buzas E. I. (2015). Biological properties of extracellular vesicles and their physiological functions. *J. Extracell. Vesicles* 4:27066. 25979354 10.3402/jev.v4.27066 PMC4433489

[B89] Yoon Y. M. Lee J. H. Song K. H. Noh H. Lee S. H. (2020). Melatonin-stimulated exosomes enhance the regenerative potential of chronic kidney disease-derived mesenchymal stem/stromal cells via cellular prion proteins. *J. Pineal Res.* 68:e12632. 31989677 10.1111/jpi.12632

[B90] Yuyama K. Sun H. Mitsutake S. Igarashi Y. (2012). Sphingolipid-modulated exosome secretion promotes clearance of amyloid-β by microglia. *J. Biol. Chem.* 287 10977–10989. 10.1074/jbc.m111.324616 22303002 PMC3322859

[B91] Zahran R. Ghozy A. Elkholy S. S. El-Taweel F. El-Magd M. A. (2020). Combination therapy with melatonin, stem cells and extracellular vesicles is effective in limiting renal ischemia–reperfusion injury in a rat model. *Int. J. Urol.* 27 1039–1049. 10.1111/iju.14345 32794300

[B92] Zhang X. Li X. Ning F. Shang Y. Hu X. (2019). TLE4 acts as a corepressor of Hes1 to inhibit inflammatory responses in macrophages. *Protein Cell* 10 300–305. 10.1007/s13238-018-0554-3 29869113 PMC6418302

[B93] Zhao D. Yu Y. Shen Y. Liu Q. Zhao Z. Sharma R. (2019). Melatonin synthesis and function: evolutionary history in animals and plants. *Front. Endocrinol.* 10:249. 10.3389/fendo.2019.00249 31057485 PMC6481276

[B94] Zhao L. Hu C. Zhang P. Jiang H. Chen J. (2019). Genetic communication by extracellular vesicles is an important mechanism underlying stem cell-based therapy-mediated protection against acute kidney injury. *Stem Cell Res. Ther.* 10:119. 30995947 10.1186/s13287-019-1227-8 PMC6471862

[B95] Zhao L. Hu C. Zhang P. Jiang H. Chen J. (2020). Melatonin preconditioning is an effective strategy for mesenchymal stem cell-based therapy for kidney disease. *J. Cell Mol. Med.* 24 25–33. 10.1111/jcmm.14769 31747719 PMC6933322

[B96] Zheng Y. Hasan A. Nejadi Babadaei M. M. Behzadi E. Nouri M. Sharifi M. (2020). Exosomes: multiple-targeted multifunctional biological nanoparticles in the diagnosis, drug delivery, and imaging of cancer cells. *Biomed. Pharmacother.* 129:110442. 10.1016/j.biopha.2020.110442 32593129

[B97] Zhou H. Ma Q. Zhu P. Ren J. Reiter R. J. Chen Y. (2018). Protective role of melatonin in cardiac ischemia-reperfusion injury: from pathogenesis to targeted therapy. *J. Pineal Res.* 64:e12471. 10.1111/jpi.12471 29363153

